# Tissue specific expression of human fatty acid oxidation enzyme genes in late pregnancy

**DOI:** 10.1186/s12944-016-0373-6

**Published:** 2016-11-21

**Authors:** Jose L. Bartha, Fernando Bugatto, Álvaro Fernández-Deudero, Rosa Fernández-Macías, Germán Perdomo

**Affiliations:** 1 Department of Obstetrics and Gynecology, University Hospital “La Paz”, Madrid, Spain; 2 Division of Maternal and Fetal Medicine, Department of Obstetrics and Gynecology, University Hospital “Puerta del Mar”, Cádiz, Spain; 3 University Hospital “Puerta del Mar”, Research Unit, Cádiz, Spain; 4 University of Burgos, School of Nursery, Burgos, Spain

**Keywords:** Fatty acid metabolism, Long-chain acyl CoA dehydrogenase, Medium-chain acyl CoA dehydrogenase, Placenta

## Abstract

**Background:**

Abnormal fatty acid oxidation (FAO) is associated with maternal and fetal complications during pregnancy. The contribution of maternal and fetal tissues to FAO capacity during late pregnancy is important to understand the pathophysiology of pregnancy-associated complications. The aim of this study was to determine the expression levels of mitochondrial FAO enzymes in maternal and fetal tissues during late normal pregnancy.

**Methods:**

We have measured by Real-time PCR the levels of long- and medium -chain acyl-CoA dehydrogenase (LCHAD and MCAD), two acyl-CoA dehydrogenases that catalyze the initial step in the mitochondrial FAO spiral.

**Results:**

LCHAD and MCAD were expressed in maternal skeletal muscle, subcutaneous adipose tissue, placenta, and maternal and fetal blood cells. LCHAD gene expression was four- to 16-fold higher than MCAD gene expression in placenta, adipose tissue and skeletal muscle. In contrast, MCAD gene expression was ~5-fold higher in fetal blood than maternal blood (*p* = 0.02), whereas LCHAD gene expression was similar between fetal blood and maternal blood (*p* =0.91).

**Conclusions:**

LCHAD and MCAD are differentially expressed in maternal and fetal tissues during normal late pregnancy, which may represent a metabolic adaptation in response to physiological maternal dyslipidemia during late pregnancy.

## Background

A hallmark of late normal pregnancy is a physiological hyperlipidemic state characterized by maternal hypertriglyceridemia and elevated plasma free fatty acids (FFA) levels [1, 2]. In this way, maternal fat accumulated in the adipose tissue during early pregnancy, become available for placental transfer during the last trimester of pregnancy, satisfying the exacerbated fetal demand for fatty acids [1, 3, 4]. It has been proposed that mitochondrial fatty acid oxidation (FAO) in placenta is an important energy source for survival, function and growth of both the placenta and the fetus [5–7].

FAO capacity in maternal and fetal tissues has not been extensively investigated in normal late pregnancy. Furthermore, there are no previous data comparing the expression levels of FAO enzymes in the placenta with other maternal and fetal tissues. Therefore, the aim of this study was to analyse FAO gene expression and protein levels in different tissues during late pregnancy. To this end, we analysed the expression levels of long- and medium -chain acyl-CoA dehydrogenase (MCAD and LCHAD respectively), two acyl-CoA dehydrogenases that catalyze the initial step in the mitochondrial FAO spiral.

## Methods

### Study group and tissue collection

This is a prospective study performed from pregnancies monitored at the Department of Obstetrics and Gynecology, University Hospital “Puerta del Mar” (HUPM). Twelve healthy pregnant women at term who were going to have an elective Caesarean section (without labour) for clinical reasons were recruited from our maternity ward for the study. Characteristics of the study group are shown in Table 1. Reasons for Caesarean section included: previous Caesarean section because of cephalopelvic disproportion (*n* = 6), breech presentation (*n* = 4), previous poor perinatal outcome (*n* = 1) and extreme myopia (*n* = 1). Specific exclusion criteria included women with history of LCHAD deficiency, preeclampsia, HELLP syndrome or acute fatty liver disease in previous pregnancies, history of chronic hypertension or other co-morbid disease.

**Table 1:** Demographics of the women and newborns participating in the study

	Median	Percentile 25	Percentile 75
Gestational age at delivery (weeks)	39,00	38,00	40,00
Maternal weight (Kg)	67,00	59,25	82,75
Maternal height (m)	1,62	1,57	1,65
Body mass index (Kg/m^2^)	26,66	22,82	32,23
Maternal age (years)	33	29	34
Birth weight (g)	3160,00	2900,00	3720,00

During Caesarean section, 6 mL of maternal blood, samples of subcutaneous fat tissue and rectoabdominal striated muscle were taken. After placenta delivery, samples of the maternal side of the placenta and 6 mL of fetal blood from the umbilical vein were also taken. Placental samples were obtained from at least four different portions after careful examination to remove contaminating fragments of maternal decidua, and pooling them together for the gene expression analysis. All tissues were washed with ice-cold PBS to remove residual blood, and samples were snap-frozen in liquid nitrogen and stored at −80 °C. Leukocytes were isolated from blood samples using the Red Blood Cell Lysis Buffer Kit (Roche) following manufacturer’s instructions, and samples were store at −80 °C.

### Quantitative real-time PCR

Total RNA was extracted from fetal and maternal blood, placenta, subcutaneous fat tissue and skeletal muscle by using the High Pure RNA Isolation kit (Roche) following the manufacturer’s protocol. This procedure included an on-column DNase I digestion step. cDNA synthesis was carried out using random hexamer primers and the components from the Transcriptor First Strand cDNA Synthesis kit (Roche). LCHAD, MCAD and an internal reference β-actin transcript were quantified according to a fluorescence-based real-time detection method performed with an ABI PRISM 7000 sequence detection system (Applied Biosystems). The real-time quantitative PCR reaction was performed from cDNA using TaqMan® Universal PCR Master Mix, No AmpErase® UNG (Applied Biosystems). PCR was performed with annealing at 55 °C for 30 s, extension at 72 °C for 90 s, and denaturation at 94 °C for 30 s and 25 cycles. Primer sequences and target-specific fluorescence labelled TaqMan probes were obtained from TaqMan® Gene Expression Assays (Applied Biosystems). Assay references were as follows: Hs00426191_m1 (amplicon length 134 bp) for LCHAD, Hs00163494_m1 (amplicon length 117 bp) for MCAD and Hs99999903_m1 (amplicon length 171 bp) for β-actin. PCRs were performed in 96-well microtiter plates, according to the manufacturer’s instructions (Applied Biosystems). Relative gene expression levels were calculated by using the 2 ^-ΔΔCT^ method [8]. Data are presented as the LCHAD and MCAD gene expression normalized to the β -actin gene and relative to placental sample. Two independent analyses were performed for each sample and for each gene [9].

### Immunoblot analyses

To determine tissue protein levels, aliquots (100 mg) of placenta or skeletal muscle tissue were homogenized in 1 mL of ice-cold PBS, pH 7,5, supplemented with 2 μl of protease inhibitor cocktail (Sigma, Madrid, Spain) and 1 mM phenylmethylsulfonyl fluoride (Sigma). The tissue lysates were sonicated three times for 10 s each on ice. Afterwards, the lysates were subjected to centrifugation at 18,000 *X g* for 30 min at 4 °C, and the protein concentration of the supernatant was measured by the Bradford method. Aliquots of 20 μg of protein extracts per lane were applied to 10% SDS polyacrylamide gels. Proteins were transferred onto polyvinylidene difluoride (PVDF) membranes for immunoblotting by conventional means, using a polyclonal anti-LCHAD antibody (1:500 dilution; Abcam, Cambridge, UK), polyclonal anti-MCAD antibody (1:200 dilution; ab23675, Abcam), and anti-actin (1:2000 dilution, Sigma). Signals were detected by chemiluminescence, and bands within the linear range were quantified using the NIH Image software.

### Statistical analysis

Distribution of variables was checked using both the histogram and the Kolmogorov-Smirnov test. Since most of variables followed a non-normal distribution, numerical data are shown as median and interquartile range. Comparisons in FAO gene expression between LCHAD and MCAD, and between the placenta and the different tissues were done by using the Wilcoxon Signed Ranks Test as well as the median and 95% confidence interval method. Statistical significance was previously set to the 95% level (*p* < 0.05).

## Results

MCAD and LCHAD gene expression in the different tissues are shown in Table 2. As expected, MCAD and LCHAD were expressed in three important maternal tissues involved in the regulation of FAO, such as skeletal muscle, adipose tissue and placenta. Interestingly, these genes were also expressed in both fetal and maternal blood cells (Table 2).

**Table 2:** MCAD and LCHAD gene expression^a^

	Median	Percentile 25	Percentile 75
MCAD			
Placenta	0,0057	0,0047	0,0138
Fetal blood	0,0015	0,0011	0,0038
Maternal blood	0,0007	0,0002	0,0013
Maternal subcutaneous fat	0,0098	0,0053	0,0183
Maternal striated muscle	0,0190	0,0019	0,0759
LCHAD			
Placenta	0,1277	0,0950	0,1767
Fetal blood	0,0048	0,0038	0,0079
Maternal blood	0,0063	0,0020	0,0093
Maternal subcutaneous fat	0,0538	0,0349	0,1366
Maternal striated muscle	0,0552	0,0359	0,1339

^a^Values are given in URA (units relative to β-actin)

When analysing LCHAD and MCAD gene expression in fetal and maternal blood cells, we found that MCAD gene expression was ~5-fold higher in fetal blood than maternal blood (median 4.55 95% CI 2.07–16.00) (*p* = 0.02). In contrast, LCHAD gene expression ratio was similar between fetal blood cells and maternal blood cells (median 0.84 95% CI 0.40–5.17) (*p* = 0.91).

**Fig. 1:**
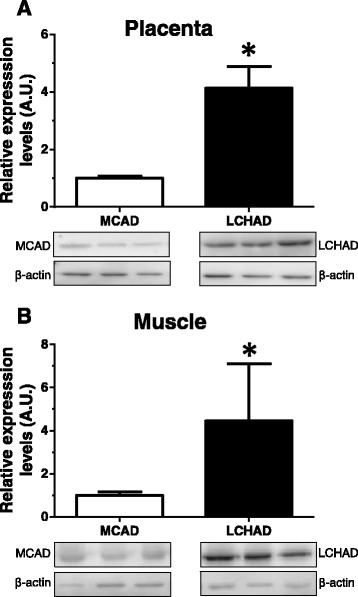
**a**, **b** Expression levels of LCHAD and MCAD in maternal tissues. Western blot analyses of LCHAD and MCAD protein levels were carried out in placenta (*n* = 12) and skeletal muscle (*n* = 12) from pregnant women. Frozen placental and skeletal muscle tissues were used to quantify placental LCHAD and MCAD content (see Methods for details). β-actin expression was determined to ensure similar protein loading. Top: the y-axis represents the relative protein expression levels of MCAD vs. LCHAD in arbitrary units (A.U.). Bottom: a representative picture of the western blot is shown. Data are means ± SEM for 12 independent experiments.*p < 0.05 relative to MCAD by unpaired Student’s *t*-test

A detailed analysis of the LCHAD relative expression to MCAD in each tissue revealed that LCHAD gene expression was 16-fold higher than MCAD gene expression in placenta (Table 3). In addition, LCHAD expression was ~4-fold higher than MCAD in subcutaneous fat, striated muscle and maternal blood cells; whereas LCHAD expression was ~2-fold higher than MCAD expression in fetal blood cells (Table 3). Western blot analyses in maternal tissues confirmed gene expression data. As shown in Fig. 1, LCHAD protein expression was four-fold higher than MCAD protein expression in placenta (Fig. 1a) and skeletal muscle (Fig. 1b) tissues.

**Table 3:** LCHAD relative to MCAD expression in each tissue

	Median	95% CI	*p* = ^1^
Placenta	16,06	11.80–29.04	0.002
Fetal blood	2,60	1.09–8.68	0.01
Maternal blood	4,06	2.71–24.00	0.008
Maternal subcutaneous fat	4,56	2.57–9.06	0.003
Maternal striated muscle	3,85	1.21–25.11	0.02

^1^Based on Wilcoxon Signed Ranks Test, differences between LCHAD and MCAD gene expression in each tissue

Finally, we examined the relative MCAD and LCHAD gene expression levels in each tissue relative to placental tissue. MCAD expression levels were significantly higher in placenta than in fetal and maternal blood cells, but similar than in maternal subcutaneous fat and striated muscle (Table 4). On the other hand, LCHAD expression levels were significantly higher in placenta than in fetal and maternal blood cells, and maternal subcutaneous fat. In addition, LCHAD expression levels were significantly higher in placenta than in striated muscle although this difference did not achieve statistical significance.

**Table 4:** MCAD and LCHAD gene expression relative to placenta

		Median	95% CI	*p* = ^1^
MCAD	Fetal blood	0.22	0.13–0.27	0.003
	Maternal blood	0.05	0.04–0.09	0.008
	Maternal subcutaneous fat	1.71	0.41–2.18	0.11
	Maternal striated muscle	1.39	0.35–10.67	0.15
LCHAD	Fetal blood	0.03	0.02–0.08	0.002
	Maternal blood	0.04	0.01–0.10	0.002
	Maternal subcutaneous fat	0.54	0.25–0.88	0.003
	Maternal striated muscle	0.52	0.28–1.07	0.22

^1^Based on Wilcoxon Signed Ranks Test, differences in gene expression between the placenta and the rest of the studied tissues

## Discussion

In this study, we have used gene expression and western blot analyses to investigate the mitochondrial FAO capacity of maternal and fetal blood. We took advantage of comparing different tissues from the same pregnant women avoiding using tissues from non-pregnant women. Overall, we found a good correlation between gene expression levels and protein expression levels in maternal placenta and skeletal muscle tissues. In the case of placental tissue, LCHAD protein levels were 4-fold higher than MCAD. The discrepancies between data from gene expression levels (16-fold higher than MCAD) and protein levels may be explain in two ways: 1) the sensitivity of the PCR analysis is higher than western blot, accounting for the differences in expression levels; and 2) LCHAD might be post-transcriptionally regulated in human placenta.

Given the paucity, and sometimes, contradictory studies on the physiological and pathophysiological regulation of placental fatty acid metabolism, our study point out the relevance of fatty acid metabolism during late pregnancy [10–13]. The observation that LCHAD expression is higher in placenta than in maternal skeletal muscle underline the energetic demand of this tissue during late pregnancy, and its specificity regarding medium- or -long fatty acid metabolites. In addition, LCHAD is one of the two acyl-CoA dehydrogenase that carries out the third step in the β-oxidation of fatty acids with chain lengths of C12 to C18 [6]. LCHAD deficiency is an inborn error associated with reduced FAO capacity and pregnancy-associated complications such as preeclampsia [6]. From a mechanistic point of view, a deficiency of LCHAD would lead to the accumulation of toxic metabolites such as hydroxyacylcarnitines. These harmful metabolites inhibit mitochondrial FAO enzymes, uncouple oxidative phosphorylation, and impair ATP production, leading to reduced mitochondrial FAO capacity [14–17]. In addition, these metabolites would increase placental lipid peroxidation [18], and might be transferred to maternal circulation contributing to endothelial damage and hypertension [6, 19]. Following this rationale is plausible to hypothesize that elevated levels of LCHAD in placenta would enhance placental FAO, avoiding accumulation of harmful metabolites, and protecting maternal and fetal tissues. Thus, our observations that LCHAD expression is elevated in placenta might reflect a physiological response to maternal dyslipidemia.

Due to ethical reasons, we have used leukocytes isolated from blood to analyse the expression levels of LCHAD and MCAD in fetal tissues. Leukocytes are able to β-oxidize short- and long -chain fatty acids [20]. We found that comparing fetal with maternal blood cells, a much higher MCAD expression was found in fetal blood. These observations suggest that fetal leukocytes preferentially use medium-chain fatty acids rather than long-chain fatty acids as an energy source. Medium-chain fatty acids in fetal circulation might come from the placenta, where elevated levels of LCHAD transform long-chain fatty acids into medium-chain fatty acids. Thus, the differential expression of mitochondrial genes in maternal and fetal tissues would contribute to a more efficient utilization of fatty acids as an energy source. Nonetheless, the contribution of blood cells to the overall fatty acid metabolism in the fetus remains to be deciphered.

We acknowledge that a limitation of this study is the small sample size. Thus, our results may not be considered representative of the general population. Further studies including greater sample size are warranted to generalize our conclusions.

## Conclusion

In conclusion, LCHAD and MCAD are differentially expressed in maternal and fetal tissues during normal late pregnancy, which may represent a metabolic adaptation in response to physiological maternal dyslipidemia during late pregnancy.

### Key message box

- Abnormal fatty acid oxidation (FAO) is associated with maternal and fetal complications during pregnancy.
- LCHAD and MCAD are differentially expressed in maternal and fetal tissues during normal late pregnancy, which may represent a metabolic adaptation in response to physiological maternal dyslipidemia during late pregnancy.

## Abbreviations

FAO: Fatty acid oxidation
FFA: Free fatty acids
HELLP syndrome: Hemolysis, elevated liver enzymes, and low platelets syndrome
LCHAD: Long-chain L-3-hydroxyacyl-CoA dehydrogenase
MCAD: Short-chain acyl-CoA dehydrogenase
